# Prevalence and genetic characterization of viral gastroenteritis in hospitalized children aged <5 years in Yunnan Province, China, 2020–2022

**DOI:** 10.3389/fped.2024.1497467

**Published:** 2025-01-08

**Authors:** Nan Li, Enfa Qiao, Zhaojun Duan, Lili Li, Lili Jiang, Jianping Cun, Xiaofang Zhou, Zhi Chao Wang, Yongming Zhou, Yihui Cao

**Affiliations:** ^1^ Yunnan Provincial Key Laboratory of Public Health and Biosafety & Institute for AIDS/STD Control and Prevention, Yunnan Center for Disease Control and Prevention, Kunming, Yunnan, China; ^2^ National Institute for Viral Disease Control and Prevention, China CDC, Beijing, China; ^3^ Department of Acute Infectious Diseases Control and Prevention, Yunnan Center for Disease Control and Prevention, Kunming, Yunnan, China

**Keywords:** acute gastroenteritis, rotavirus, norovirus, human enteric adenovirus, human astrovirus, sapovirus

## Abstract

**Background:**

Rotavirus (RV), norovirus (NoV), human enteric adenovirus (HAdV), human astrovirus (HAstV), and sapovirus (SaV) are important viral causes of acute gastroenteritis (AGE) in children. However, limited information is available regarding AGE in Yunnan, Southwest China.

**Methods:**

To investigate the prevalence of group A rotavirus (RVA), norovirus genogroups I (GI) and II (GII), and HAdV, HAstV, and SaV in children aged <5 years hospitalized with AGE between 2020 and 2022.

**Results:**

Stool samples were collected from 612 children hospitalized with AGE. A total of 266 of the 612 children presented with AGE (43.46%; 266/612). RVA was detected in 28.76% (176 of 612) of the children. Rotavirus G9P[8] was the most frequent genotype in 2020 and 2021. In 2022, G8P[8] became the dominant genotype combination circulating in Yunnan Province. The norovirus positivity rate was present in 11.93% (73/612) of the 612 samples. Of the 45 GII successfully sequenced samples, GII.4 was the dominant genotype, accounting for 51.11% (23 of 45), followed by GII.3 [P12] (28.89%; 13 of 45). The positivity rates for SaV, HAstV, and HAdV were 2.94% (18/612), 3.43% (21/612), and 4.74% (29/612), respectively. HAdV-F41 was the predominant genotype and non-enteric HAdV-C2 and HAdV-A12 were also observed in Yunnan. Male children had a higher incidence of AGE than female children upon infection with RV, NoV, and HAdV. The highest incidence of AGE was observed among children aged between 12 and 23 months (62.50%; 120/192), followed by children aged between 24 and 35 months (52.44%; 43/82). The incidence rate of the infection peaked (78.62%; 125/159) in the first 3 months of the year, followed by the next 3 months (66.67%; 70/105).

**Conclusions:**

RV and NoV remained the most important agents causing AGE. RV G8P[8] became the dominant circulating genotype instead of G9P[8] in Yunnan in 2022. The authors suggest that monitoring should be strengthened to prevent outbreaks caused by RV G8P[8]. New vaccines, such as the RV G8P[8] genotype, should be considered.

## Introduction

Acute gastroenteritis (AGE) is a common illness that affects people of all ages and remains a leading cause of morbidity and mortality (1–3). Young children are especially vulnerable to severe disease (4, 5). AGE poses a significant burden on public health (6–8). Bacterial gastroenteritis among children has been decreasing in China (9). Viral etiologies such as group A rotavirus (RVA), norovirus (NoV), human enteric adenovirus (HAdV), human astrovirus (HAstV), and sapovirus (SaV) play a more important role in children aged <5 years, particularly in infants and young children aged <2 years (10), than in children of other age groups (11–13). To better understand the incidence of AGE among children aged <5 years in Yunnan Province, Southwest China, and to characterize the epidemiology of viral gastroenteritis, we analyzed surveillance data collected by AGE sentinel hospitals in Yunnan Province during the period between 2020 and 2022.

## Materials and methods

### Sample collection

Fecal samples were collected from hospitalized children aged <5 years who sought medical care for AGE at the national viral AGE sentinel hospitals in Yunnan between 2020 and 2022. In total, 612 fecal specimens were collected from these children. In accordance with the National Surveillance Protocol for Viral Diarrhea (2021 version), AGE was defined as more than three episodes of abnormal stools (liquid, watery, mucous, or bloody purulent) in a 24-h period or less than three abnormal stools per day along with vomiting. Information on the fecal specimens, such as the sample collection date, and personal information such as age and gender, were collected from the patients, with the consent of their guardians obtained for the personal information. The stool samples were stored at room temperature for no more than 4 h. All fecal specimens were stored at −70°C before screening.

### DNA and RNA extraction

The stool samples were suspended in 10% phosphate-buffered saline, mixed for 10 s, and then centrifuged at 8,000×*g* for 5 min. Viral DNA and RNA were extracted from 200 μl of the supernatant using the Viral Nucleic Acid Isolation Kit (BioPerfectus, Beijing, China, SDK60104) according to the manufacturer's instructions.

### Detection and phylogenetic analysis of RV, NoV, and HAdV

All stool samples were screened for rotavirus (RV), NoV, SaV, HAstV, and HAdV by real-time PCR with the RVA/NoV (GI and GII) Nucleic Acid Testing Kit (ABT Beijing, China) and the Sapovirus/Astrovirus/Enteric Adenovirus Nucleic Acid Testing Kit (ABT, Beijing, China) according to the manufacturer's instructions. Both kits contained an internal amplification control. In the PCR assays, an external positive and an external negative control were used to ensure the accuracy of the screening results. Positive RVA, NoV, and HAdV samples with Ct <30 were genotyped by the Sanger sequencing method. The one-step RT-PCR Kit (Qiagen, Germany, 210212) was used to amplify the partial sequences of the VP7/VP4 genes with 881 bp/663 bp RT-PCR products against RVA. The VP7 sequences have been submitted to GenBank (accession numbers: PQ048122-PQ048188). Some positive samples with low nucleic acid load were genotyped using the Rotavirus Fluorescent Genotyping Kit (ABT, China, A9369YH-25T). For norovirus-positive samples, the partial sequences of regions B and C with the primer MON432/G1SKR for GI and the primer MON431/G2SKR for GII yielded 579 and 570 bp RT-PCR products, respectively. The norovirus sequences determined in this study have been deposited in GenBank (accession numbers: PQ044503-PQ044541). In the same way, the hexon gene of HAdV was analyzed to determine the HAdV genotype. The HAdV sequences have been submitted to GenBank under accession number PQ048098-PQ048121. The aforementioned genotype-specific primers are listed in Table 1. Phylogenetic trees of RVA, NoV, and HAdV were constructed using MEGA software (version 11) along with the neighbor-joining and Kimura two-parameter methods. Chi-square tests were used to compare proportions, and *p*-values <0.05 were considered statistically significant.

**Table 1:** The genotype-specific primers used in the study.

Virus	Primer sequences	Sequences (5′–3′)	Sizes (bp)
Rotavirus G-typing (14)	F-VP7	ATGTATGGTATTGAATATACCAC	881
	R-VP7	AACTTGCCACCATTTTTTCC	
Norovirus GII (15)	F-MON431	TGGACIAGRGGICCYAAYCA	570
	R-G2SKR	CCRCCNGCATRHCCRTTRTACAT	
Norovirus GI (15)	F-MON432	TGGACICGYGGICCYAAYCA	579
	R-G1SKR	CCA ACCCARCCATTRTAC A	
Adenovirus (16)	F-Hexon	TTCCCCATGGCICAYAACAC	482
	R-Hexon	CCCTGGTAKCCRATRTTGTA	

## Results

### Basic information

**Figure 1:**
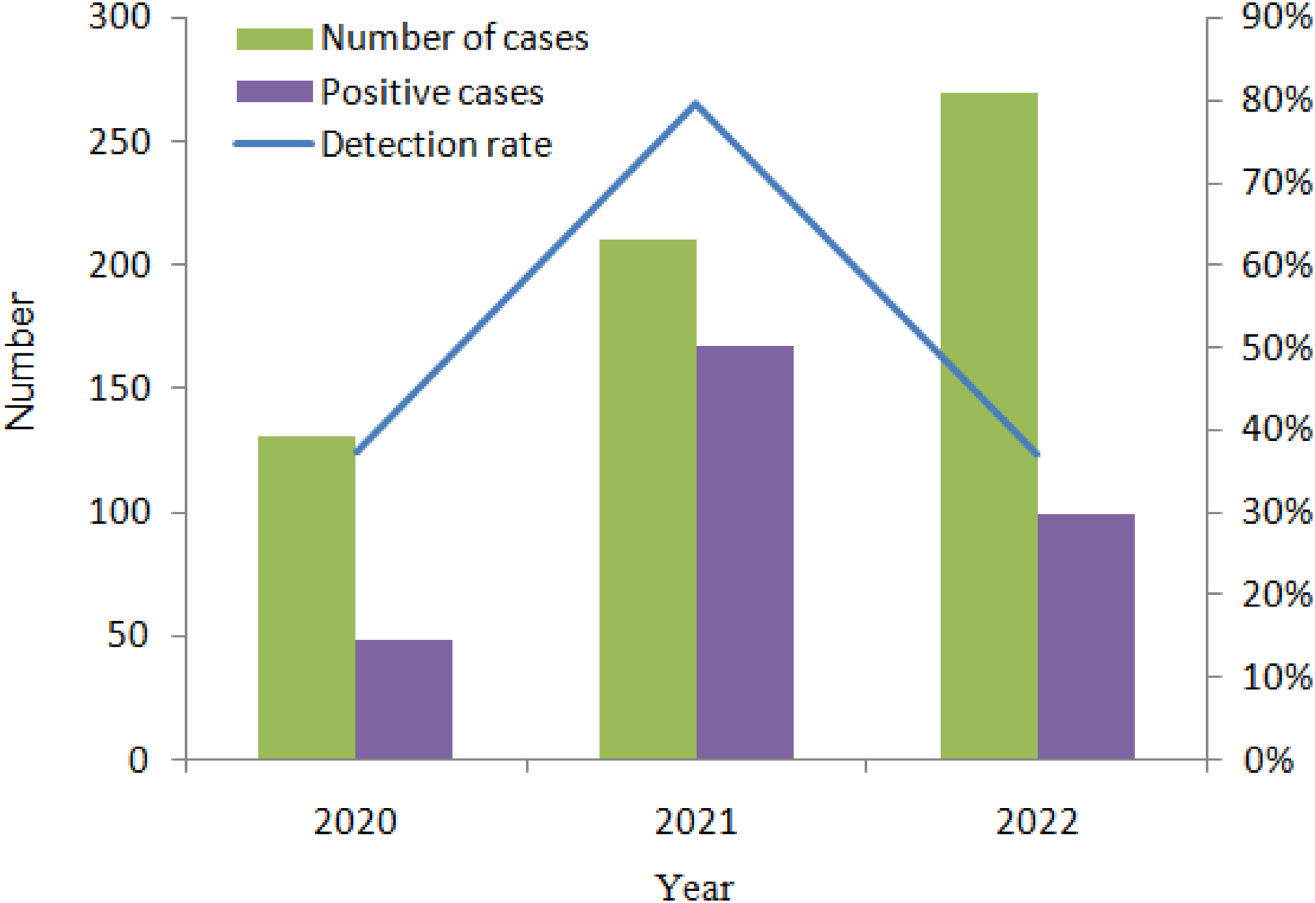
The number of diarrhea cases and the distribution of virus-positive cases, 2020–2022.

In total, 807 children aged between 0 and 59 months (mean age, 17.35 months) were hospitalized with AGE in Yunnan Province, Southwest China, between January 2020 and December 2022. Of these children, 612 (75.84%) were enrolled in our study, and their stool samples were submitted to the laboratory. The number of stool samples in each year from 2020 to 2022 was 131, 211, and 270, respectively (Figure 1). Of the 612 patients, there were 372 boys and 240 girls, and the count of male patients was 1.55 times more than that of females. The average patient age was 20 months (median age 12 months); 266 samples (43.46%) displayed at least one of the viral etiology positivity rates.

In total, 612 samples were collected for three consecutive monitoring years: 2020, 2021, and 2022. There were 270 samples in 2022 and 131 samples in 2020. The highest viral detection rate was 79.62% in 2021.

### Pathogen composition

#### Rotavirus A

**Figure 2:**
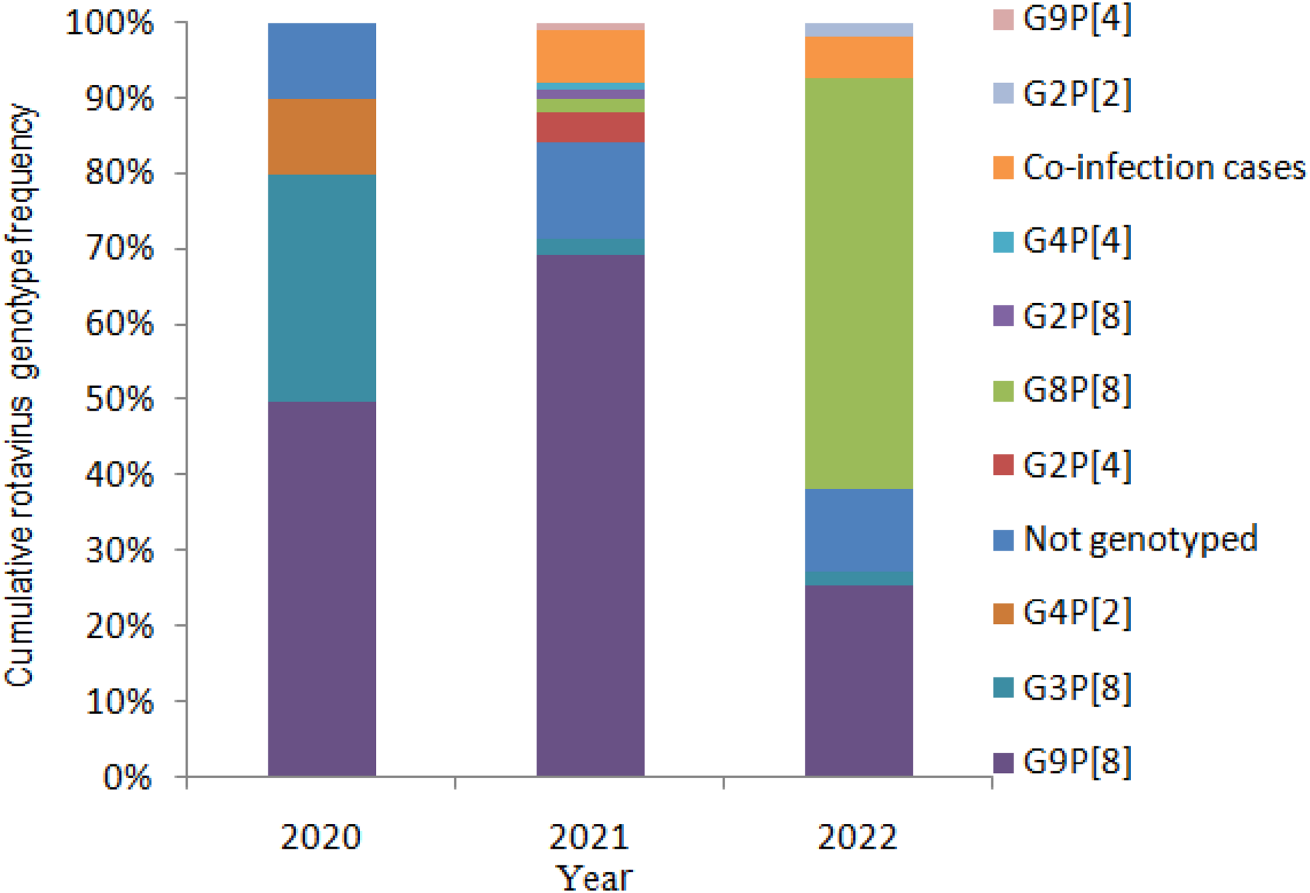
The cumulative rotavirus genotype frequency in Yunnan Province, 2020–2022.

In total, 176 rotaviruses were detected in the 612 stool samples (28.76%). In 2020, 20 cases of patients with a rotavirus positivity rate of 15.27% were reported, including 10 with G9P[8] and 6 with G3P[8]. In 2021, the RV positivity rate was 47.87% (101/211), including 70 patients with G9P[8] and 2 with G8P[8]. In 2022, the rotavirus positivity rate was 20.37% (55/270), including 30 patients with G8P[8] and 14 with G9P[8]. The G8P[8] genotype became the predominant genotype in Yunnan (Figure 2).

Rotavirus genotype diversity was found in children aged <5 years hospitalized with gastroenteritis; RV G9P[8] was the dominant genotype, circulating with G3P[8] before 2022. RV G8P[8] was first detected in Yunnan in 2021, and it became the predominant strain in 2022.

#### Phylogenetic analysis of RVA

**Figure 3:**
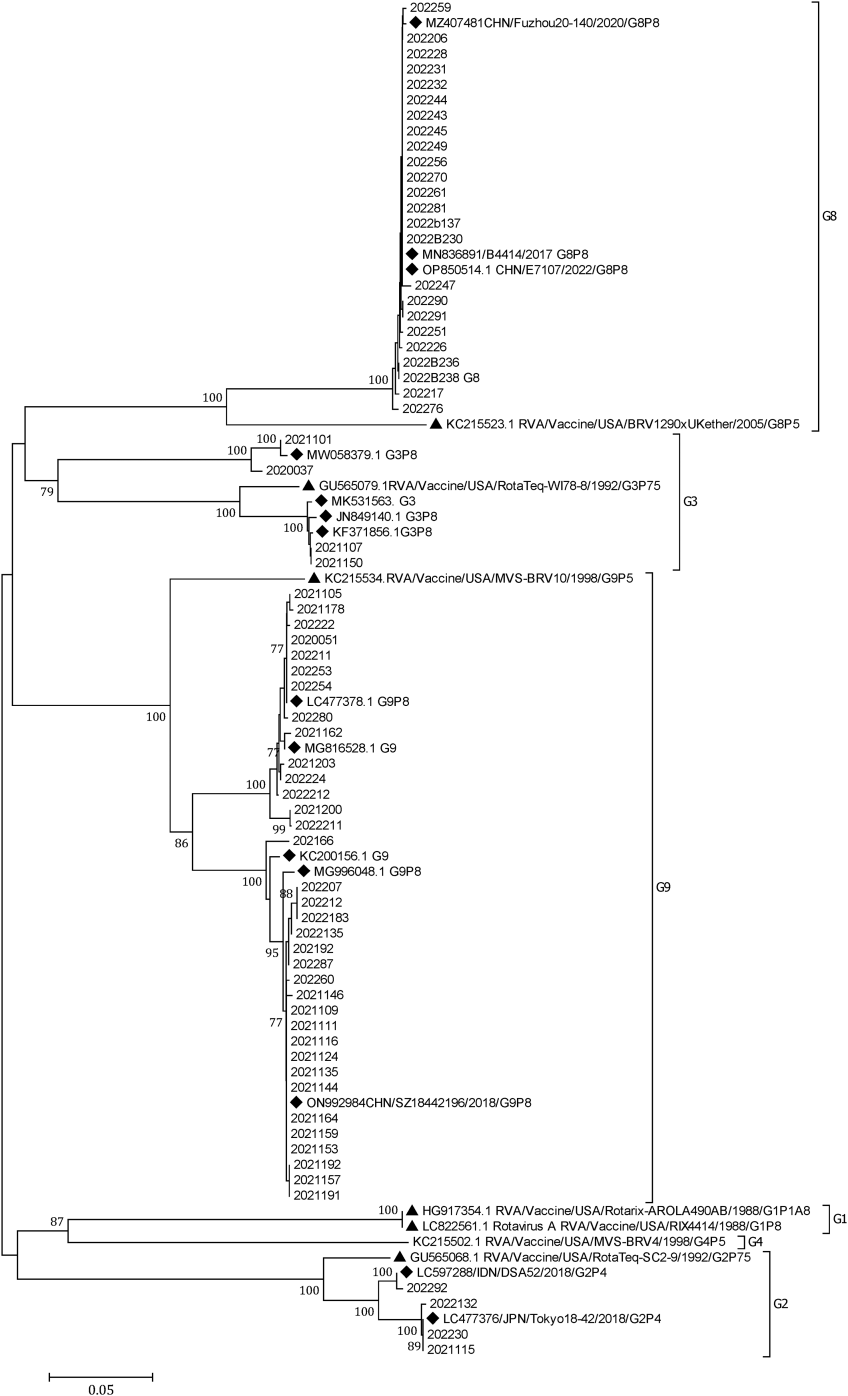
A phylogenetic tree constructed from the nucleotide sequences of the partial RV VP7 gene. A phylogenetic analysis based on the nucleotide sequences of the partial (756 bp) VP7 gene of RVA strains detected in children hospitalized with AGE in Yunnan, 2020–2022. G9 and G8 were the predominant G genotypes. A phylogenetic tree was constructed by the neighbor-joining method using the Kimura two-parameter method. Closely related reference strains from GenBank are indicated by accession numbers. Bootstrap support >75% is shown. A GenBank database (reference strains) (◆). Vaccine strains (▴).

A phylogenetic tree was constructed from the nucleotide sequences of the partial RVA VP7 gene. It showed that G8 and G9 were the prevalent genotypes in Yunnan (Figure 3).

#### Norovirus

**Figure 4:**
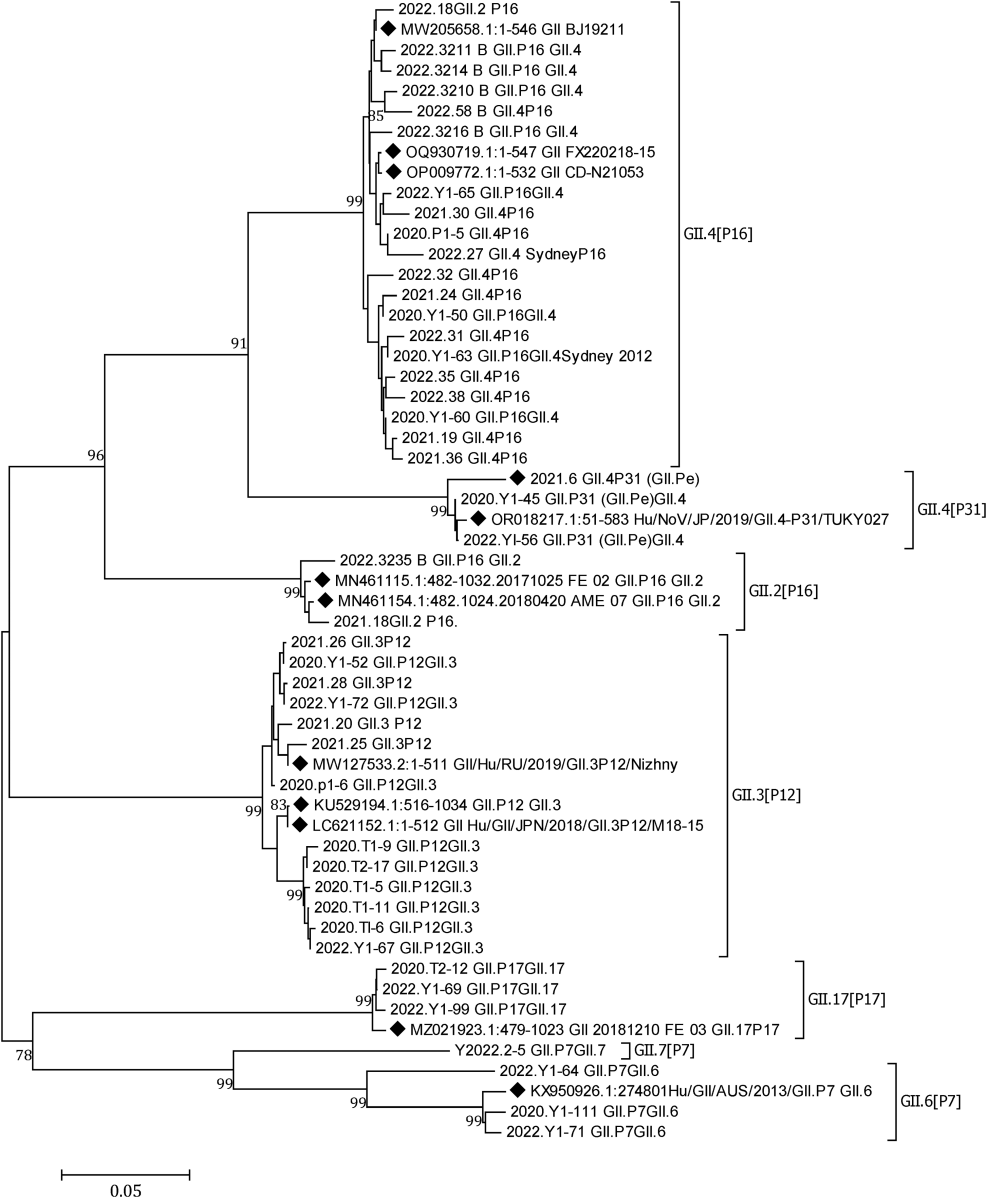
A phylogenetic tree constructed from norovirus GII nucleotide sequences. A neighbor-joining phylogenetic tree for the partial capsid gene (GII-576 bp) of NoV strains detected in children hospitalized with AGE in Yunnan, 2020–2022. Closely related reference strains from GenBank are indicated by accession numbers. Bootstrap support >75% is shown. The scale bar represents 0.05 nucleotide substitutions per site across the indicated region. A GenBank database (reference strains) (◆).

Norovirus was detected in 73 (11.93%) of the 612 patient samples. The positivity rates in 2020, 2021, and 2022 were 9.16% (12/131), 12.80% (27/211), and 12.59% (34/270), respectively. Of the 73 norovirus-positive samples, there were 5 GI and 68 GII. In total, 45 GII samples were successfully genotyped, with the breakup being 20 GII.4 [P16], 13 GII.3 [P12], 3 GII.4 [P31], 3 GII.17 [P17], 3 GII.6 [P7], 2 GII.2 [P16], and 1 GII.7 [P7]. GII.4 [P16] and GII.3 [P12] were the dominant genotypes circulating in Yunnan (Figure 4).

#### SaV, HAstV, and HAdV

**Figure 5:**
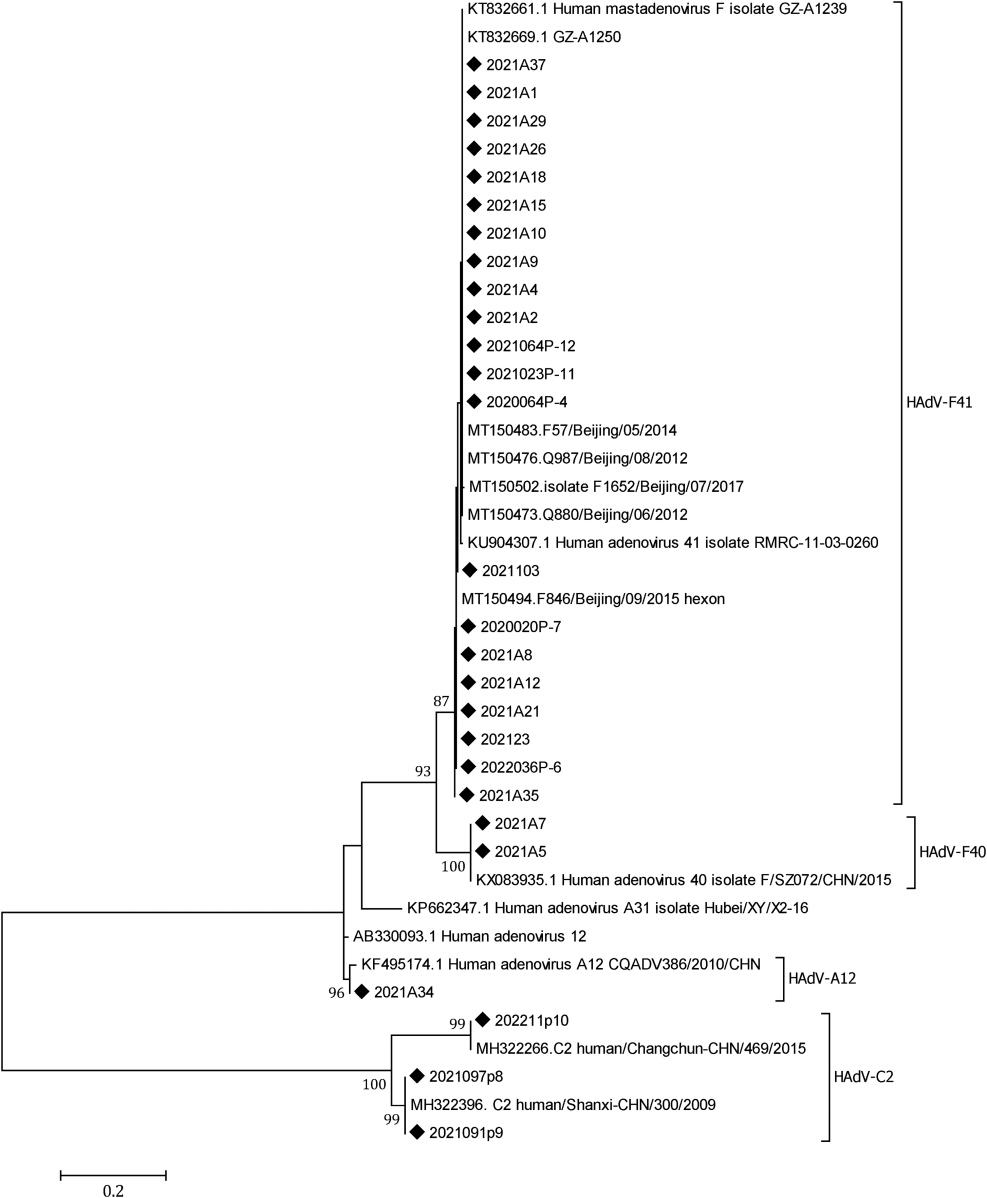
A phylogenetic tree of the HAdV hexon genes. A phylogenetic tree of HAdV hexon gene nucleotide sequences was constructed using the neighbor-joining method. HAdV-F41 was the dominant genotype circulating in Yunnan, 2020–2022. Closely related reference strains from GenBank are indicated by accession numbers. Bootstrap support >75% is shown. The scale bar represents 0.2 nucleotide substitutions per site across the indicated region. The HAdV strains detected in the study (◆).

A total of 18 SaV, 21 HAstV, and 29 HAdV were detected in the 612 samples; the positivity rates were 2.94% (18/612), 3.43% (21/612), and 4.74% (29/612), respectively. A total of 27 of the 29 HAdVs were successfully sequenced, revealing 21 (77.78%) HAdV-F41, 3 (11.11%) HAdV-C2, 2 (7.41%) HAdV-F40, and 1 (3.70%) HAdV-A12. HAdV-F41 was the predominant genotype circulating in Yunnan (Figure 5).

### Prevalence and distribution of coinfection

One virus was detected in 43.46% (266/612) of the samples (i.e., positive samples). Two viruses were present in 43 patients, and three viruses were simultaneously present in 4 patients. Thus, coinfection was detected in 7.68% (47/612) of patients. Both norovirus and rotavirus were screened in 24 samples (51.06%; 24/47).

### Epidemiological features

The detection rates of RVA, NoV, SaV, HAdV, and HAstV in male patients were 29.30% (109/372), 14.25% (53/372), 2.15% (8/372), 4.84% (18/372), and 2.69% (10/372), respectively, and the detection rates in female patients were 27.92% (67/240), 8.33% (20/240), 4.17% (10/240), 4.58% (11/240), and 4.58% (11/240), respectively. Except for cases of SaV and HAstV infection, male children had a higher incidence of AGE than their female counterparts; the incidence of AGE significantly differed with respect to NoV infection (*χ*^2^ = 4.86, *P* = 0.028).

The highest rate of incidence of AGE was observed among children aged between 12 and 23 months (62.50%; 120/192), followed by children aged between 24 and 35 months. The lowest incidence rate was observed in children aged between 48 and 59 months (8.00%; 4/50) (Table 2). The detection rates of mixed infections in male and female children were 4.41% (27/612) and 2.61% (16/612), respectively. The detection rates of positive cases significantly differed among seasons (*χ*^2^ = 179.25, *P* < 0.001). The highest detection rate (78.62%; 125/159) was observed in the first quarter of the year, followed by the second quarter (66.67%; 70/105) (Table 2).

**Table 2:** Detection of viral gastroenteritis in children in Yunnan Province between 2020 and 2022.

Characteristic		Cases (*n*)	Positivity rate (%)							
			RVA	NoV	SaV	HAdV	HAstV	Dual infection	Triple infection	Total
Season	Spring (Jan–Mar)	159	61.01	25.16	3.77	0	5.66	14.47	1.26	78.62
	Summer (Apr–Jun)	105	40.95	17.14	4.76	13.33	5.71	11.43	1.90	66.67
	Autumn (Jul–Sep)	72	5.56	2.78	2.78	4.17	1.39	1.39	0	15.28
	Winter (Oct–Dec)	276	11.59	4.71	1.81	4.35	1.81	2.54	0	21.74
	*χ* ^2^		146.934	48.632	3.161^a^	24.348^a^	6.870^a^	28.606	5.265^a^	179.25
	*P*		<0.001	<0.001	0.357	<0.01	0.061	<0.001	0.074	<0.001
Age (months)	0–5	73	15.07	9.59	0	4.11	1.37	2.74	0	27.40
	6–11	144	22.22	9.03	2.78	6.25	2.78	2.78	0	40.28
	12–23	192	43.75	26.04	4.69	5.21	5.21	14.06	0.52	62.50
	24–35	82	40.24	13.41	2.44	3.66	2.44	7.32	1.22	52.44
	36–47	71	22.54	7.04	2.82	4.23	4.23	4.23	1.41	33.80
	48–59	50	0	2.00	2.00	2.00	2.00	2.00	2	2.00
	*χ* ^2^		57.548	16.516	3.971^a^	1.529^a^	2.901^a^	23.380	4.593^a^	76.945
	*P*		<0.001	0.006	0.532	0.920	0.712	<0.001	0.316	<0.001
	Female	240	27.92	8.33	4.17	4.58	4.58	6.67	0	42.92
	Male	372	29.30	14.25	2.15	4.84	2.69	7.26	1.08	44.09
Gender	*χ* ^2^		0.136	4.857	1.481	0.021	1.581	0.078	2.598	0.081
	*P*		0.712	0.028	0.224	0.885	0.209	0.780	0.107	0.776

^a^ Fisher's exact test.

## Discussion

This 3-year investigation systematically explored viral agents causing AGE among children aged <5 in Yunnan Province, southwest China, between the years 2020 and 2022. The surveillance showed that RVA, NoV, and HAdV were the most common causes of AGE. It ' reported that approximately 60% of hospital admissions for AGE worldwide are attributed to RVA infection, which tends to have a more severe course and is even significantly associated with mortality (17–19). RVA genotypes vary in different regions (20–22). It has been reported that rotavirus G1 is the most widespread genotype globally and constitutes the dominant genotype in many regions (20, 22, 23). G9P[8] was the predominant RVA genotype in China in 2016–2018 (24). In our study, 73 of 612 (11.92%) stool specimens demonstrated RVA positivity. Rotavirus G9P[8] was the dominant epidemic genotype, circulating with G3P[8] in 2020–2021 in Yunnan. RVA G8P[8] became the predominant epidemic strain in 2022. The tourism industry in Yunnan is well-developed due to the unique geographical conditions and excellent climate. In combination with a large population (48 million), these factors enable the new strain to circulate easily. Vaccination is an effective method against RVA infection, and the authors suggest that a vaccine that includes G8P[8] should be developed as early as possible against a potential rotavirus outbreak. China's National Immunization Program has achieved remarkable success, but it does not include the rotavirus vaccine; moreover, Yunnan is a relatively underdeveloped province. Both factors contribute to the low vaccination rate.

NoV and SaV are members of the Caliciviridae family. NoV is the most common cause of sporadic viral AGE and gastroenteritis outbreaks (25–27) and exhibits a highly diverse genotype, which is divided into 10 distinct geno groups (GI-GX). These groups are subclassified into genetic clusters or genotypes (28). In our study, 73 (11.93%) NoVs and 18 (2.94%) SaVs were detected in 612 patients, and NoV GII was the most prevalent geno group (93.15%; 68/73). GII.4 was identified as the predominant genotype in all settings (29–33). In this study, we obtained similar results: GII.4 [P16] was predominant in Yunnan during 2020–2022, followed by GII.3[P12]. No norovirus-targeting medications are available, and vaccines are still in development. Currently, the prevention of NoV infection relies on frequent hand hygiene, limited contact with virus-infected individuals, and disinfection of contaminated environmental surfaces (34).

HAdVs, one of the major etiologic agents associated with AGE in young children (35, 36), are classified into six subgenera (A–G). Types 40 (HAdV-F40) and 41 (HAdV-F41) in subgenus F are causative agents of enteric infections (37). Non-enteric genotypes are significantly associated with AGE among children aged <5 years of age (38). Two non-enteric HAdV serotypes were detected in our study, HAdV-C2 and HAdV-A12. HAdV infection was identified in 4.74% of the 612 tested children. Among the 27 sequenced HAdVs, HAdV-F41 was the predominant genotype (77.78%), followed by HAdV-C2 (11.11%), HAdV-F40 (7.41%), and HAdV-A12 (3.70%). There is a need to closely monitor AGE caused by non-enteric HAdV genotypes other than HAdV-F40 and HAdV-F41.

In terms of coinfection, RVA and NoV were the main pathogens of AGE in hospitalized children with a significant difference (*χ*^2^ = 28.61, *P* < 0.05). Three pathogens were even observed in four patients. Because of the limited number of cases, the clinical data were insufficient to establish a direct relationship with the severity of clinical symptoms. Viral infections are more common during the cold season (4, 39), but the seasonality differs among regions (40, 41). The highest detection rate (78.62%; 125/159) in Yunnan was observed during the first 3 months of the year (*χ*^2^ = 179.25, *P* < 0.001), followed by the next 3 months (66.67%; 70/105). Perhaps this is related to Yunnan's unique geographical environment and climate.

Children <5 years of age are susceptible to AGE, especially those aged between 12 and 23 months (*χ*^2^ = 76.945, *P* < 0.001), and the incidence rate of AGE was 62.50% (120/192) in this age group, followed by 52.44% (43/82) in the 24–35-month age group.

In this study, only a statistically significant association related to gender was determined for NoV infection (*p* = 0.028). Table 2 shows that NoVs were detected in 14.25% of the boys and 8.33% of the girls. For RVA infection, it was observed that boys were more infected than girls (29.30% and 27.92%, respectively), with no statistical significance, while for HAstV and SaV infections, it was observed that girls were infected more frequently than boys; however, these results were not statistically significant (*p* = 0.209 and *p* = 0.224, respectively).

During the COVID-19 pandemic, China adopted strict prevention and control policies, which objectively contributed to a reduction in diarrhea cases. In 2020, there were only 131 patients with a relatively low positivity rate (37.40%; 49/131). However, in 2021, the number of AGE cases rose rapidly. There were 211 samples with the highest incidence rate of 79.62% (168/211). In 2022, a total of 270 samples were tested, and the detection rate was 37.04% (100/270), which showed that the incidence rate decreased almost to the same level as in 2020.

## Conclusions

This study provides baseline data concerning the possible role of gastroenteritis-causing viruses in children. The results offer valuable information that may alert healthcare providers to periods of increased likelihood of community infection. Caution should be exercised when outbreaks are caused by the new G8[P8] RVA strains and it is recommended that susceptible children actively get vaccinated against RVAs. Further long-term and large-scale epidemiological surveys are recommended to better understand the disease burden, etiologic agents, and clinical impact in Yunnan Province.

## Data Availability

The datasets presented in this study can be found in online repositories. The names of the repository/repositories and accession number(s) can be found in the article/Supplementary Material.
