# Supplementary figures and images for: Prevalence and genetic characterization of viral gastroenteritis in hospitalized children aged <5 years in Yunnan Province, China, 2020–2022
Source: Front Pediatr. 2025 Jan 8;12:1497467. doi: 10.3389/fped.2024.1497467 (PMC11750815; doi:10.3389/fped.2024.1497467)

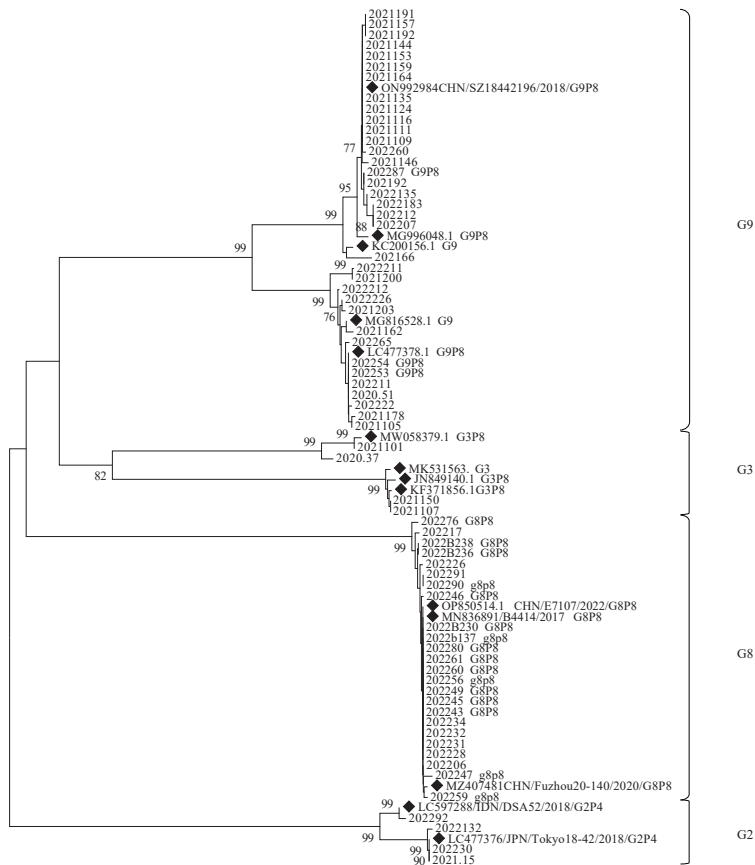

Supplement: Supplementary file 1 [file Datasheet1.pdf]
